# Developmental origins for semilunar valve stenosis identified in mice harboring congenital heart disease-associated *GATA4* mutation

**DOI:** 10.1242/dmm.036764

**Published:** 2019-06-24

**Authors:** Stephanie LaHaye, Uddalak Majumdar, Jun Yasuhara, Sara N. Koenig, Adrianna Matos-Nieves, Rahul Kumar, Vidu Garg

**Affiliations:** 1 Center for Cardiovascular Research, Nationwide Children's Hospital, Columbus, OH 43205, USA; 2 The Heart Center, Nationwide Children's Hospital, Columbus, OH 43205, USA; 3 Department of Molecular Genetics, The Ohio State University, Columbus, OH 43210, USA; 4 Department of Pediatrics, The Ohio State University, Columbus, OH 43210, USA

**Keywords:** GATA4, Valve development, Aortic valve stenosis, Genetics, Congenital heart disease

## Abstract

Congenital heart defects affect ∼2% of live births and often involve malformations of the semilunar (aortic and pulmonic) valves. We previously reported a highly penetrant *GATA4* p.Gly296Ser mutation in familial, congenital atrial septal defects and pulmonic valve stenosis and showed that mice harboring the orthologous G295S disease-causing mutation display not only atrial septal defects, but also semilunar valve stenosis. Here, we aimed to characterize the role of Gata4 in semilunar valve development and stenosis using the *Gata4^G295Ski/wt^* mouse model. GATA4 is highly expressed in developing valve endothelial and interstitial cells. Echocardiographic examination of *Gata4^G295Ski/wt^* mice at 2 months and 1 year of age identified functional semilunar valve stenosis predominantly affecting the aortic valve with distal leaflet thickening and severe extracellular matrix (ECM) disorganization. Examination of the aortic valve at earlier postnatal timepoints demonstrated similar ECM abnormalities consistent with congenital disease. Analysis at embryonic timepoints showed a reduction in aortic valve cushion volume at embryonic day (E)13.5, predominantly affecting the non-coronary cusp (NCC). Although total cusp volume recovered by E15.5, the NCC cusp remained statistically smaller. As endothelial to mesenchymal transition (EMT)-derived cells contribute significantly to the NCC, we performed proximal outflow tract cushion explant assays and found EMT deficits in *Gata4^G295Ski/wt^* embryos along with deficits in cell proliferation. RNA-seq analysis of E15.5 outflow tracts of mutant embryos suggested a disease state and identified changes in genes involved in ECM and cell migration as well as dysregulation of Wnt signaling. By utilizing a mouse model harboring a human disease-causing mutation, we demonstrate a novel role for GATA4 in congenital semilunar valve stenosis.

This article has an associated First Person interview with the joint first authors of the paper.

## Abstract (precis)

**Summary:** Cellular and molecular characterization of a mutant mouse, harboring a human disease-causing GATA4 variant, identifies cellular deficits in endothelial-to-mesenchymal transition and proliferation that cause abnormal valve remodeling and resultant stenosis.

## INTRODUCTION

Cardiac valve malformations are the most common type of congenital heart defect (CHD) affecting 1-2% of newborn infants, when including bicuspid aortic valve (BAV) (Fahed et al., 2013). Morphologic abnormalities affecting the aortic and pulmonic (semilunar) valves that result in narrowing or stenosis are some of the most prevalent types of CHD. As an isolated disease, valve defects account for nearly 10% of CHD, but a valve malformation is present in over 50% of all cases of CHD (Go et al., 2013). Congenital semilunar valve stenosis is not only a risk factor for bacterial endocarditis, but the stenosis is often progressive and results in ventricular hypertrophy and ultimately heart failure without intervention (Maganti et al., 2010). Limited treatment options exist to treat valve stenosis, and surgical or catheter-based intervention with valve replacement are ultimately required to treat the late stages of disease (Zajarias and Criber, 2009). Despite the high prevalence, the etiology of congenital semilunar valve stenosis is largely unexplored, but population-based studies support a strong genetic component for aortic and pulmonic valve stenosis, with increased relative risks of recurrence (Øyen et al., 2009).

Although it has been established that genetics play a role, a paucity of genes have been linked to non-syndromic aortic or pulmonic valve stenosis in either humans or mice (LaHaye et al., 2014). We were the first to suggest that point mutations in *GATA4* were linked to pulmonic valve stenosis when we reported a disease-segregating mutation in *GATA4* in a large family that displayed autosomal dominant fully penetrant cardiac septal defects along with partially penetrant congenital pulmonic valve stenosis (Garg et al., 2003). This mutation altered a highly conserved glycine residue to a serine at codon 296, which disrupted DNA binding ability and led to the loss of interaction with TBX5, a critical cardiac transcription factor (Garg et al., 2003). Since then, additional human genetic studies, by us and others, have demonstrated that mutations in *GATA4* are associated with pulmonic valve stenosis that occurs in conjunction with atrial septal defects, or as part of tetralogy of Fallot, supporting a potential role for this transcription factor in semilunar valve disease (Okubo et al., 2004; Sarkozy et al., 2005; Nemer et al., 2006; Tomita-Mitchell et al., 2007). To determine the mechanism by which the p.Gly296Ser mutation causes CHD, an *in vivo* mouse model was generated using homologous recombination with the orthologous p.Gly295Ser mutation knocked into the endogenous *Gata4* locus. Although the homozygous *Gata4^G295Ski/ki^* mice were embryonic lethal at E11.5, the heterozygous *Gata4^G295Ski/wt^* mice were viable and born in proper Mendelian ratios. The *Gata4^G295Ski/wt^* mice exhibited small atrial septal defects, which were attributed to the diminished expression of GATA4 target genes and functional deficits in cardiomyocyte proliferation (Misra et al., 2012). Partially penetrant semilunar valve stenosis was also reported, but not fully characterized (Misra et al., 2012). The observation of pulmonic valve stenosis in a mouse model harboring a human disease-causing *GATA4* mutation further supported a role for GATA4 in valve development, but the mechanistic basis for this association has not been investigated.

GATA4 belongs to a family of transcription factors among which GATA4, GATA5 and GATA6 are expressed in the developing heart and are proposed to cooperatively regulate developmental processes (Molkentin, 2000; LaForest and Nemer, 2011; Zhou et al., 2012; Prendiville et al., 2014). GATA4 has been extensively studied by us and others and has been shown to have critical roles from early specification of the cardiomyocyte lineage to function of the adult heart (Molkentin et al., 1997; Aries et al., 2004; Watt et al., 2004; Zeisberg et al., 2005; Rivera-Feliciano et al., 2006; Bisping et al., 2006; Heineke et al., 2007; Zhao et al., 2008; Maitra et al., 2009; Nadeau et al., 2010; Moskowitz et al., 2011; Misra et al., 2014; Yamak et al., 2014). Much of this work has focused on the role of GATA4 in cardiomyocyte differentiation and myocardial development, along with endocardial cushion development and atrioventricular (AV) valve formation, in which endothelial loss of GATA4 prevents endothelial-to-mesenchymal transition (EMT), resulting in hypocellular AV valves and lethality between embryonic day (E)11.5 and E12.5 (Rivera-Felicano et al., 2006). Although the cellular contributions to the AV cushions and/or valves are primarily from EMT-derived cells, the development of the outflow tract (OFT) cushions and/or valves also involves contributions from neural crest-derived cells and second heart field (SHF)-derived cells (McCulley et al., 2008; High et al., 2009; Hinton and Yutzey, 2011). GATA4 is expressed in endocardial cells, along with EMT-derived cells, in the developing OFT and has demonstrated roles in SHF-derived cells in normal heart development (Rojas et al., 2008).

Development of the cardiac valves is an intricate process that involves several cell lineages, which are tightly controlled though multiple genetic programs (Combs and Yutzey, 2009; Hinton and Yutzey, 2011; Lincoln and Garg, 2014). Semilunar valve development commences with the formation of the OFT endocardial cushions in the wall of the linear heart tube. Subsequently, EMT is initiated by signals from the adjacent myocardium and launches the invasion and transformation of endothelial cells to mesenchymal cells. These mesenchymal cells proliferate and lead to the expansion and elongation of the valve primordia. In addition to EMT, cardiac neural crest cells (CNC) also contribute to the development of the OFT. The OFT cushions can be divided into two portions, the proximal (p)OFT, which comprises mostly cells of EMT descent, and the distal (d)OFT, which is mostly composed of CNC-derived mesenchymal cells. The pulmonic and aortic valves each contain three cusps. The pulmonic valves are made up of the anterior cusp, the right cusp and the left cusp, whereas the aortic valve is named in association with the location of the coronary arteries and is composed of the right coronary cusp (RCC), the left coronary cusp (LCC) and the non-coronary cusp (NCC). The valve primordia develop by a process of thinning, reshaping, elongation and remodeling of the extracellular matrix (ECM). Valve remodeling occurs well into postnatal development, eventually forming a stratified trilaminar structure composed of organized ECM layers of elastin, collagen and proteoglycans (Wirrig and Yutzey, 2011; Lin et al., 2012).

Here, we aim to describe the development of the semilunar valve stenosis phenotype in *Gata4^G295Ski/wt^* mice and to determine the underlying mechanisms. Examination of the semilunar valves of *Gata4^G295Ski/wt^* mice led to the identification of functionally and structurally stenotic valves with thickened, dysmorphic leaflets. These abnormalities were noted soon after birth, consistent with congenital disease, and analysis of the embryonic valve primordia demonstrate abnormal remodeling of the OFT cushions with associated molecular changes consistent with abnormal valve maturation. Additional evidence supports that these developmental changes are the result of abnormal development of the endocardial cushions (Lin et al., 2012). Our work is the first to mechanistically link a *GATA4* human disease-causing mutation with congenital semilunar valve stenosis.

## RESULTS

### Mice heterozygous for the *Gata4* p.Gly295Ser mutation display congenital semilunar valve stenosis

**Fig. 1.**
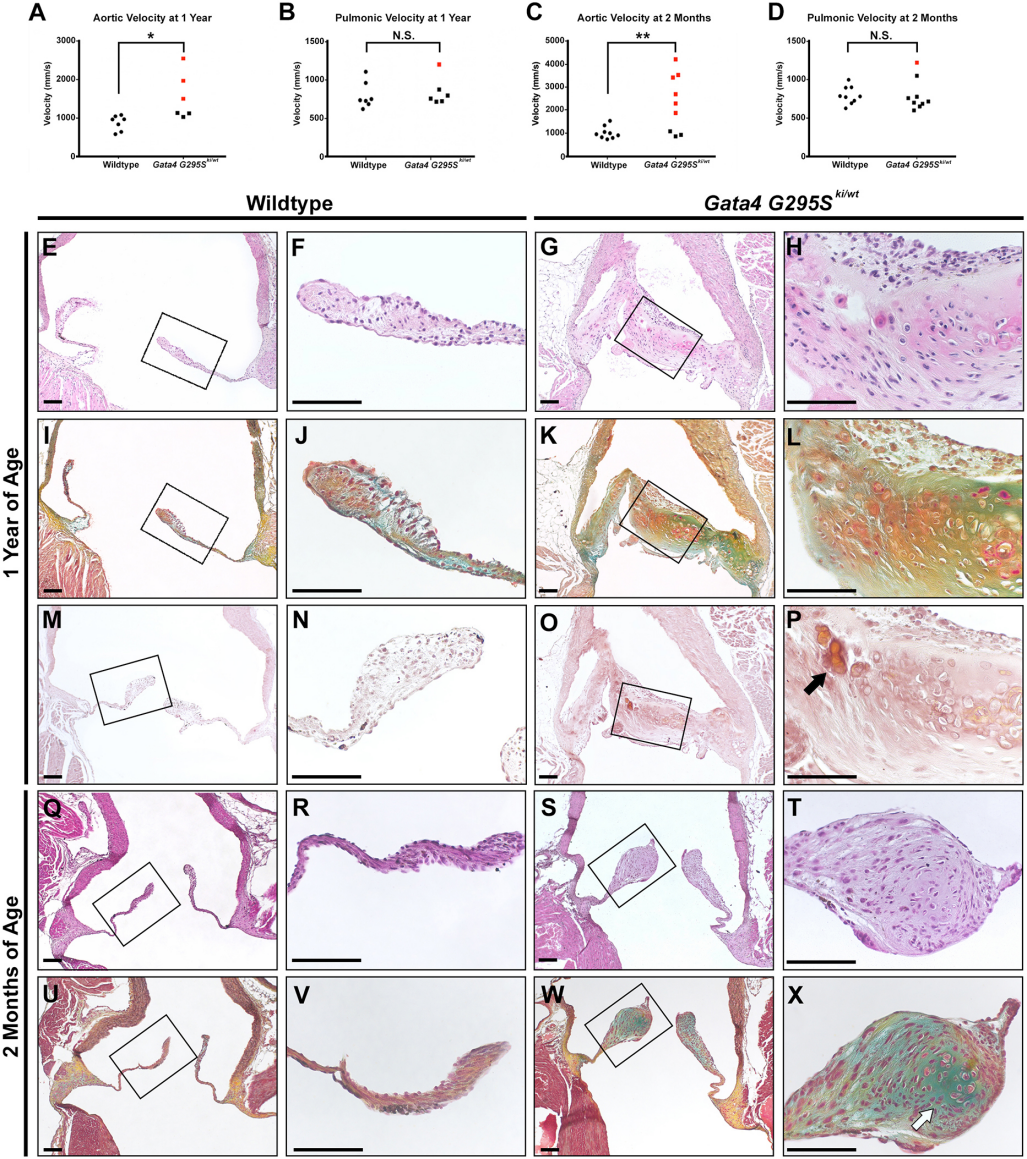
***Gata4^G295Ski/wt^* mice exhibit semilunar valve stenosis.** (A-D) Valve stenosis is found at 1 year (A,B) and 2 months of age (C,D) in *Gata4^G295Ski/wt^* mice compared with wild-type controls as determined by echocardiography (1 year old mice: *n*=7 for wild type and *n*=5 for *Gata4^G295Ski/wt^*; 2 month old mice: *n*=9 for wild type and *Gata4^G295Ski/wt^*). Velocity from *Gata4^G295Ski/wt^* mice indicated as squares, wild-type mice are shown as circles. Red circles or squares indicate stenosis. (E-L) Histological sections of aortic valve of *Gata4^G295Ski/wt^* mice exhibit thickened, dysmorphic leaflets (G,H) and abnormal ECM, as shown by Russell-Movat's Pentachrome staining (K,L) at 1 year of age, compared with wild-type control valves (E,F,I,J). F,H,J and L are high magnification images of the boxed areas in E,G,I and K, respectively. (M-P) Positive Alizarin red staining consistent with a calcific nodule (black arrow) is noted at 1 year of age (O,P) compared with wild-type control (M,N). P and N are high magnification images of the boxed areas in O and M, respectively. (Q-X) Aortic valve sections from 2 month old *Gata4^G295Ski/wt^* mice demonstrate thickened, dysmorphic leaflets (S,T) and abnormal ECM (W,X) compared with wild-type control valves (Q,R,U,V). White arrow highlights proteoglycan-rich nodule formation. R,T,V, and X are high magnification images of the boxed areas in Q,S,U and W, respectively. For each histological section shown in E-X, *n*=3 for wild type and *n*=3 for *Gata4^G295Ski/wt^*. For Russell-Movat's Pentachrome stains, yellow indicates elastin and blue indicates proteoglycans. *P≤0.05; **P≤0.005; N.S., *P* >0.05 (Student's t-test). Scale bars: 100 µm.

We previously described mice harboring the human disease-causing mutation in *Gata4* (*Gata4^G295Ski/wt^*) (Misra et al., 2012). Phenotypic characterization of adult mice harboring a single mutant allele identified highly penetrant atrial septal defects along with semilunar valve stenosis, although at a lower penetrance. To further characterize the semilunar valve disease in this model, echocardiography was performed on 1 year old *Gata4^G295Ski/wt^* mice along with wild-type littermates. The velocity across the aortic valve was significantly increased in *Gata4^G295Ski/wt^* mice compared with wild-type littermates, indicative of functional stenosis (*P*-value=0.0186, Fig. 1A). We did not find a significant difference between the velocity across the pulmonic valve between *Gata4^G295Ski/wt^* mice and controls (Fig. 1B). To define the onset of valve disease, we performed echocardiography on 2 month old mice and found a similar increase in the aortic velocity of *Gata4* mutant mice (*P*-value=0.0068, Fig. 1C). Again, no difference was noted across the pulmonic valve at 2 months of age (Fig. 1D). Analysis of left ventricular ejection fraction did not identify statistically significant differences in left ventricular ejection fraction, end systolic volume or diastolic volume in *Gata4^G295Ski/wt^* mice at either 2 months or 1 year of age when compared with controls (Fig. S1).

Based upon the echocardiographic studies, in which the aortic valve was predominantly affected, we focused on histological analysis of this semilunar valve. Examination of the aortic valve leaflets from 1 year old *Gata4^G295Ski/wt^* mice identified thickened aortic valve leaflets containing hypertrophic-like cells, compared with wild-type littermate controls (Fig. 1E-H). There was disorganization of collagen fibers and increased proteoglycan expression noted throughout the leaflets of heterozygote mutant mice by Russell-Movat's Pentachrome staining (Fig. 1I-L). We did identify evidence of calcific disease in one of the three *Gata4^G295Ski/wt^* valves as a nodule stained positive with Alizarin red, a marker for calcium deposits, whereas no positive staining was noted in the valves of 1 year old control littermates (Fig. 1M-P). We found similar changes upon histological analysis of 2 month old *Gata4^G295Ski/wt^* mice. The valve leaflets were thickened with abnormal collagen and proteoglycan expression when compared with controls (Fig. 1Q-X).

**Fig. 2.**
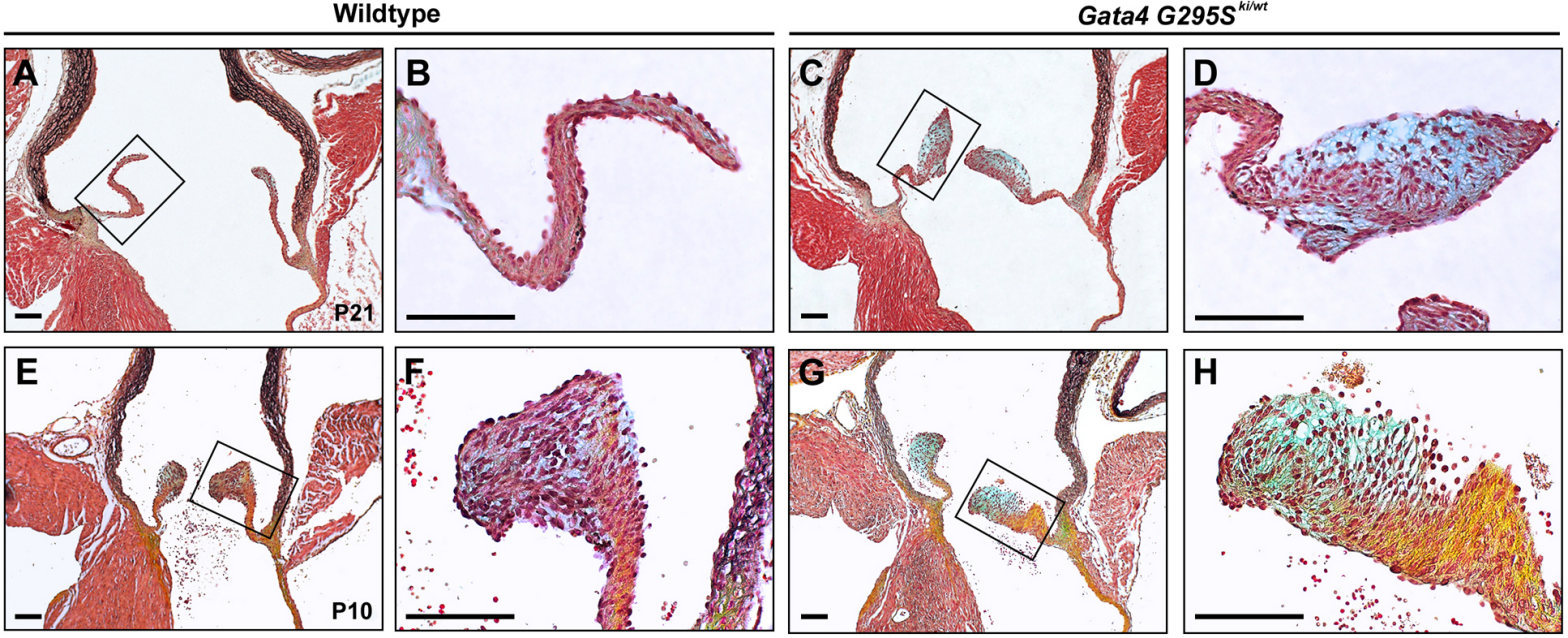
***Gata4^G295Ski/wt^* mice exhibit progressive valve disease in the postnatal period.** (A-D) Histological sections through aortic valve stained with Russell-Movat's Pentachrome show thickened leaflets with ECM disorganization and proteoglycan enrichment (blue) in *Gata4^G295Ski/wt^* valves (C,D) when compared with wild-type littermates at P21 (A,B). B and D are high magnification images of the boxed areas in A and C, respectively. (E-H) At P10, *Gata4^G295Ski/wt^* aortic valves (G-H) stained with Russell-Movat's Pentachrome are dysmorphic with abnormal ECM when compared with wild-type (E-F). F and H are high magnification images of the boxed areas in E and G, respectively. For P21 mice: *n*=4 for *Gata4^G295Ski/wt^*, *n*=3 for wild type; for P10 mice: *n*=5 for *Gata4^G295Ski/wt^*, *n*=3 for wild type. For Russell-Movat's Pentachrome stains, yellow indicates elastin and blue indicates proteoglycans. Scale bars: 100 μm.

To determine whether valve disease was present in the early postnatal period, similar to humans who harbored mutations in *GATA4*, we examined the aortic valves of *Gata4^G295Ski/wt^* mice at postnatal day (P)21 and P10. At P21, the normal aortic valve of wild-type mice has approached the completion of remodeling, elongation and thinning, whereas the valves of *Gata4^G295Ski/wt^* mice exhibit distal tip thickening with increased levels of proteoglycan expression in the distal portion of the leaflet (Fig. 2A-D). Examination of valves at P10, when the aortic valve is still undergoing postnatal remodeling, identified valves of *Gata4^G295Ski/wt^* mice that displayed an abnormal shape with markedly increased expression of collagen and proteoglycans (Fig. 2E-H). Taken together, our data suggest that *Gata4^G295Ski/wt^* mice display a phenotype that is consistent with congenital aortic valve stenosis, with dysmorphic valves that contain disorganized ECM and have the ability to calcify.

### Abnormalities in OFT cushion development in *Gata4^G295Ski/wt^* mice

**Fig. 3.**
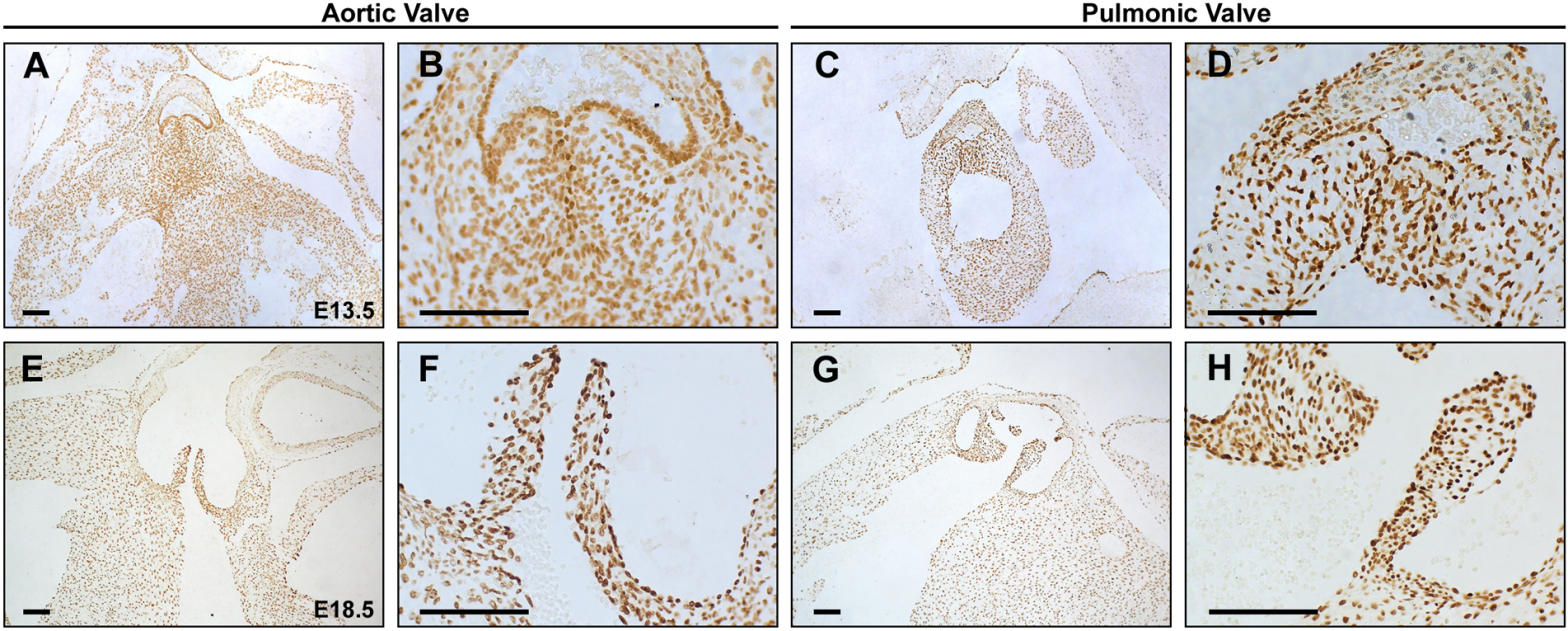
***GATA4* is expressed in the developing semilunar valves.** (A-H) Immunohistochemistry identifies GATA4 expression in the aortic (A,B,E,F) and pulmonic (C,D,G,H) valve at E13.5 (A-D) and E18.5 (E-H) of wild-type embryos. B,D,F and H are high magnification images of A,C,E and G, respectively. Scale bars: 100 μm.

The aortic and pulmonic valves are remodeled from the cardiac OFT cushions during fetal development. As GATA4 is known to be expressed in the endocardium and endocardial cushions during cardiac morphogenesis, we examined the expression of GATA4 during the early and late stages of cardiac semilunar valve remodeling (Heikinheimo et al., 1994). By immunohistochemistry, we found GATA4 expression in both the endothelial and mesenchymal/interstitial cell populations of aortic and pulmonic valves in wild-type embryos at E13.5 and E18.5 (Fig. 3A-H).

**Fig. 4.**
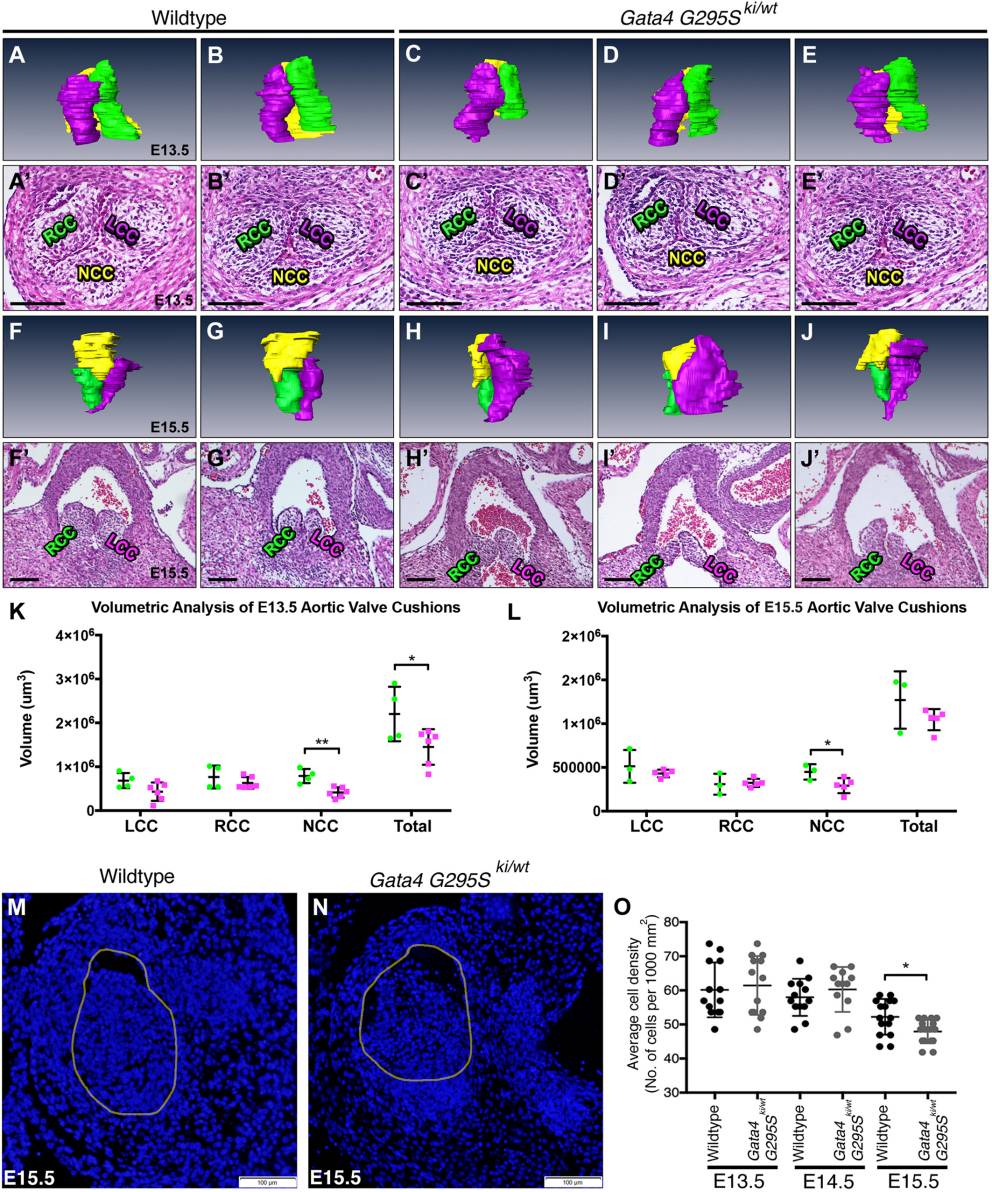
***Gata4^G295Ski/wt^* embryos display abnormalities during aortic valve cushion remodeling.** (A-E′) 3D reconstructed images of aortic cushions from histological sections of *E13.5 Gata4^G295Ski/wt^* (C′-E′) and wild-type littermate (A′,B′) embryos. *Gata4^G295Ski/wt^* reconstructed E13.5 valves (C-E) display smaller NCC (yellow) compared with wild-type littermates (A,B). (F-J) 3D reconstructed images of the aortic leaflets from histological sections of E15.5 *Gata4^G295Ski/wt^* (H′-J′) and wild-type littermates (F′,G′). *Gata4^G295Ski/wt^* reconstructed E15.5 leaflets (H-J) display smaller NCC (yellow) and abnormally shaped RCC (green) and LCC (purple) compared with wild-type littermate controls (F,G). (K) Quantification of cushion volumes (green circles, wild type; purple squares, *Gata4^G295Ski/wt^*). (L) Quantification of leaflet volumes (green circles, wild type; purple squares, *Gata4^G295Ski/wt^*). For E13.5 3D reconstructions, wild type *n*=4, *Gata4^G295Ski/wt^* *n*=6, and for E15.5 reconstructions wild type *n*=3, *Gata4^G295Ski/wt^* *n*=5. (M-O) Nuclear DAPI staining demonstrates a reduced number of nuclei per unit area in the developing aortic valve of *Gata4^G295Ski/wt^* E15.5 embryos compared with wild-type littermates, but no difference was seen at E13.5 or E14.5. Yellow circle in M and N approximates aortic lumen in representative images that were used for quantification of aortic valve cells. Wild type *n*=3, *Gata4^G295Ski/wt^* *n*=3 for each timepoint in which multiple sections from each were used for quantification. *P≤0.05; **P≤0.005 (Student's t-test). Scale bars: 100 μm.

We next determined whether the OFT cushions remodeled normally in the *Gata4^G295Ski/wt^* mice. We obtained serial sections through the length of cardiac OFT, focusing on the developing aortic valves, in E13.5 and E15.5 embryos, and performed 3D reconstruction to assess cushion volumes. At E13.5, the total volume of the aortic valve cushions was significantly smaller in the *Gata4^G295Ski/wt^* embryos compared with wild-type littermates (*P*-value=0.047; Fig. 4A-E′,K). Among the three cushions, the NCC was significantly smaller in the E13.5 *Gata4^G295Ski/wt^* embryos compared with controls (*P*-value=0.0026), whereas the RCC and LCC demonstrated a trend toward a smaller volume, but this was not statistically significant (Fig. 4A-E′,K). At E15.5, the NCC remained significantly smaller in the mutant embryos (*P*-value=0.048), but the RCC and LCC appeared to be of similar volume (Fig. 4F-J′,L). Of note, the total volume of the E15.5 aortic cushions was no longer significantly different (Fig. 4F-J′,L). Interestingly, although the RCC and LCC appear to have recovered in size at E15.5, visualization by 3D reconstruction demonstrated obvious morphological abnormalities, especially in the LCC (Fig. 4H-J). We next examined whether there were differences in proliferation or apoptosis in the OFT cushions between E13.5 and E15.5 that could be contributing to these differences. We noted a statistically significant decrease in proliferation at E13.5 in *Gata4^G295Ski/wt^* embryos, along with a small but statistically significant increase in apoptosis (Fig. S2). In addition, we assessed the cell density in the aortic valve leaflets at E15.5 and found that the *Gata4^G295Ski/wt^* embryos had decreased cell density consistent with increased production of ECM and normalization of total aortic valve cushion volume at E15.5 (Fig. 4M-O). This data also highlights the three-leaflet phenotype of the *Gata4^G295Ski/wt^* aortic valves, as no evidence of a bicuspid valve was noted. This data demonstrated smaller OFT cushions, particularly affecting the NCC, and suggested that the process of EMT along with cell proliferation may be abnormal during the early stages of OFT development in *Gata4^G295Ski/wt^* embryos.

### EMT defects in the OFT of *Gata4^G295Ski/wt^* embryos

**Fig. 5.**
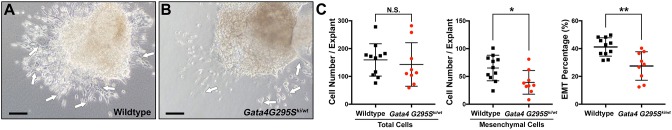
***Gata4^G295Ski/wt^ embryos* exhibit a deficit in EMT in the pOFT.** (A,B) Collagen explant assays of E10.5 pOFTs exhibit differences in EMT after 48 h of *ex vivo* culture between wild-type (A; *n*=11) and *Gata4^G295Ski/wt^* embryos (B; *n*=9). White arrows indicate mesenchymal cells. (C) Although the total number of cells is not significantly different between wild type and *Gata4^G295Ski/wt^*, the number of spindle-shaped mesenchymal cells is significantly decreased in *G295S^ki/wt^* pOFT explants when compared with wild type (*P-*value=0.018). Consistent with this, the percentage of cells having undergone EMT is also significantly less in *Gata4^G295Ski/wt^* explants compared with controls (*P*=0.0019). *P≤0.05, **P≤0.005, N.S., *P*>0.05 (Student's t-test). Scale bars: 100 μm.

As GATA4 is known to be required for normal EMT during cardiac morphogenesis, we examined EMT in the pOFT of E10.5 embryos using *ex vivo* explant assays. After 48 h of culture, pOFT explants from wild-type embryos displayed a significantly greater number of mesenchymal cells (61±22.9, *n*=11) compared with *Gata4^G295Ski/wt^* embryos (39.3±21.3, *n*=9), although the total number of cells did not significantly change (Fig. 5A-C). Mesenchymal cells were identified by the spindle-like shape acquired as endothelial cells undergo delamination and activation during the transition into a mesenchymal cell fate (Fig. 5A-C) (Zhang et al., 2014). Consistent with this, the percentage of EMT noted in control embryos (41.6%±6.6%) was significantly increased compared with *Gata4^G295Ski/wt^* (27.5%±10.2%) (Fig. 5C). In addition, the total migration distance was not significantly different between *Gata4^G295Ski/wt^* and wild-type explants (Fig. S3). Our data suggest that *Gata4* also plays an essential role in the process of EMT for pOFT cushion development, similar to its role in the atrioventricular cushions (Rivera-Feliciano et al., 2006).

### Molecular profiling of remodeling OFT cushions in *Gata4^G295Ski/wt^* embryos

**Fig. 6.**
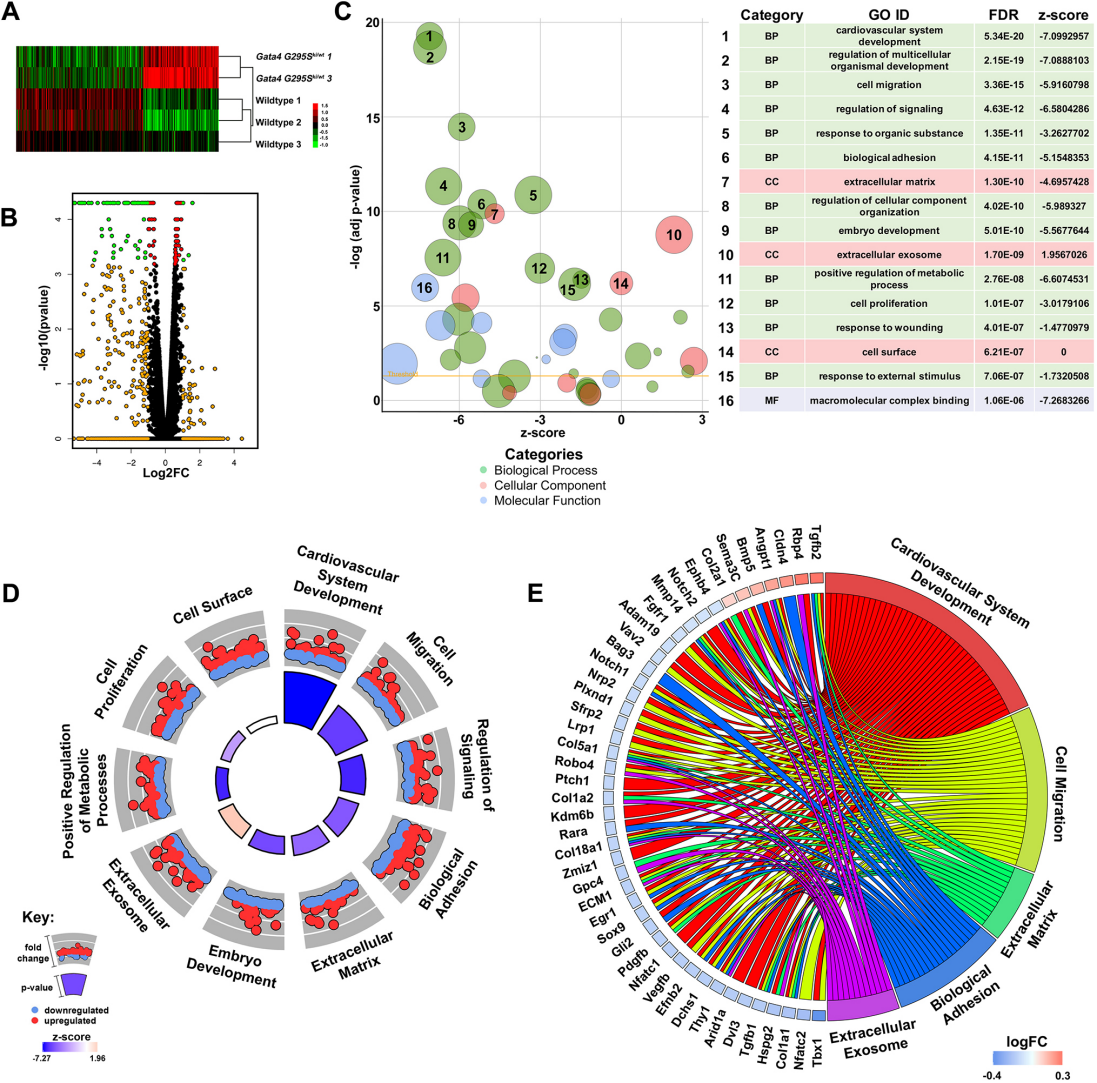
**Gene expression differences identified in E15.5 *Gata4^G295Ski/wt^* OFT.** (A) Heatmap demonstrating differential gene expression of 457 genes, with *P*-value≤0.05 and log fold change >1.25, between OFTs from E15.5 *Gata4^G295Ski/wt^* and wild-type littermates. The expression intensity is displayed as log-2-transformed and plotted on a red (upregulated) to green (downregulated) scale. (B) Volcano plot demonstrating the differential gene expression of the *Gata4^G295Ski/wt^* OFTs compared with control (red, *P*-value≤0.05; orange, expression change ≥1.5-fold; green, *P*-value≤0.05 and a fold change ≥1.5-fold). (C) Top 100 enriched GO terms, depicted as a bubble plot, with the top 16 terms labeled [green, biological process (BP); red: cellular component (CC); blue, molecular function (MF)]. (D) Circle plot highlighting ten of the top 16 GO terms. Red dots, genes within the pathway that are upregulated; blue dots, genes that are downregulated. The height of the inner circle section is associated with *P*-value (taller, more significant) and the color of the inner circle is associated with *z*-score (blue, downregulated; red, upregulated). (E) Chord plot representing 45 differentially expressed genes linked to the associated GO term(s). The logFC is represented on a scale of blue (downregulated) to red (upregulated).

**Fig. 7.**
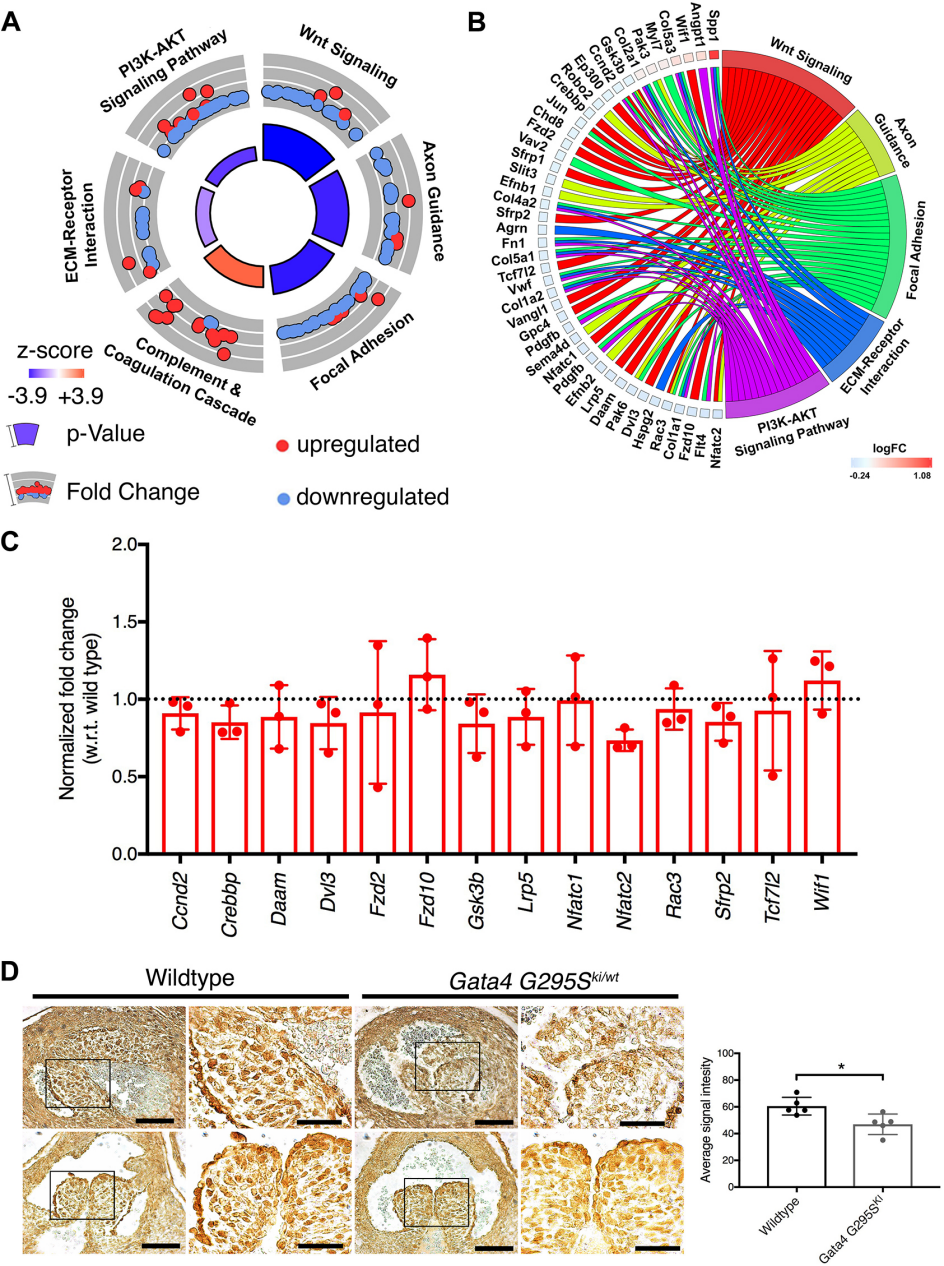
**Dysregulation of the Wnt signaling pathway identified in the E15.5 *Gata4^G295Ski/wt^* OFT.** (A) KEGG Pathway analysis identified changes in key heart development pathways, which are represented as a circle plot and includes the most enriched pathway, Wnt signaling. Red dots, genes within the pathway that are upregulated; blue dots, genes that are downregulated. The size of the inner circle section is associated with *P*-value (taller, more significant) and the color of the inner circle is associated with *z*-score (blue, downregulated; red, upregulated). (B) Chord plot highlighting 40 differentially expressed genes linked to the associated KEGG pathway(s). The log fold change (logFC) is represented on a scale of blue (downregulated) to red (upregulated). (C) Transcript levels of representative Wnt signaling pathway genes identified in RNA-seq quantified in three independent littermate samples of E15.5 *Gata4^G295Ski/^*^wt^ OFTs by RT-qPCR when compared with wild-type littermate OFTs. All genes were normalized with respect to (w.r.t.) internal *Gapdh* control. Dotted line represents wild-type expression. (D) Immunohistochemistry for β-catenin protein in E15.5 aortic valve leaflets from *Gata4^G295Ski/wt^* and wild-type littermates. An overall decrease in β-catenin expression in the endothelial lining of the cushions, as well as a loss of punctate expression within the nuclei of the interstitial cells, is noted in *Gata4^G295Ski/wt^* when compared with the littermate control. Representative images from two mutant and two wild-type (littermate) embryos are shown (right column for each genotype is magnification of boxed area in left column). Graph shows quantification of positive staining using ImageJ (*n*=5). *P≤0.05 (Student's t-test). Scale bars: 80 μm (left); 40 μm (magnifications, right).

Although the OFT cushion size appeared to be normal at E15.5 in *Gata4^G295Ski/wt^* embryos, we did note morphological abnormalities when performing the 3D reconstructions. Therefore, we asked whether there were differences in the molecular expression profiles in the OFT of *Gata4^G295Ski/wt^* OFT and wild-type littermates. We performed RNA-seq analysis using total RNA extracted from microdissected E15.5 OFT from wild-type and *Gata4* mutant embryos. We identified statistically significant differential gene expression between *Gata4^G295Ski/wt^* and wild-type littermates as shown by heatmap and volcano plot (Fig. 6A,B). As expected, the *Gata4* p.G295S mutation was present in mutant samples, whereas wild-type embryos did not have any reads to the *Gata4* p.G295S variant (Table S1). Gene ontology (GO) analysis was performed on the 1146 differentially expressed genes and identified enrichment in GO terms involved in cardiovascular system development, cell migration, biological adhesion and ECM (Fig. 6C,D). These GO terms were associated with changes in genes that have previously been associated with valve development and disease (shown in Fig. 6E). Additionally, KEGG pathway analysis was performed on this same dataset and identified Wnt as the most statistically significantly enriched KEGG pathway (Fig. 7A). In addition, five other pathways were found to be significantly enriched, including axon guidance, focal adhesion and ECM-receptor interaction pathways, which have also been associated with development of the cardiac valves (Fig. 7B). We specifically focused on the Wnt signaling pathway, which is essential for development of the OFT as it plays key roles in EMT and cardiac neural crest migration. Overall, the Wnt signaling pathway appears to be downregulated as shown by the negative *z*-score (Fig. 7A). Several genes involved in Wnt signaling were disrupted in the *Gata4^G295Ski/wt^* mouse model, and are highlighted in Fig. S4. We validated these gene expression differences by real-time quantitative PCR (RT-qPCR) using RNA extracted from E15.5 cardiac OFTs and found a similar trend for several of the genes, with downregulation of the Wnt signaling pathway highlighted by downregulation of *Nfatc2* and upregulation of *Wif1*, an inhibitor of Wnt signaling (Fig. 7C). Similar validation was found with several genes implicated in the axon guidance and focal adhesion pathways (Fig. S5). The canonical Wnt signaling pathway was examined in the developing aortic valve of E15.5 *Gata4^G295Ski/wt^* and wild-type littermates. Using immunohistochemistry, we found decreased expression of β-catenin in endothelial and interstitial cells in *Gata4^G295Ski/wt^* embryos (*n*=5, Fig. 7D and Fig. S6). In summary, these data support a state of disease in the remodeling E15.5 OFT of *Gata4* mutant mice with gene expression changes in ECM, cell proliferation and migration, along with dysregulation of Wnt signaling.

## DISCUSSION

The molecular mechanisms underlying congenital semilunar valve stenosis are not well understood. Mutations in *GATA4*, although primarily linked to cardiac septal defects, have also been reported to be associated with congenital pulmonic valve stenosis, including three unrelated familial cases with a p.Gly296Ser mutation. Here, we describe congenital semilunar valve disease in mice harboring the corresponding disease-causing mutation in *Gata4* (p.Gly295Ser). These mutant mice display histological and molecular abnormalities during semilunar valve development that are consistent with valve disease. In addition, the valve disease in the *Gata4^G295Ski/wt^* mice does not result in early lethality, allowing for the ability to follow disease progression. These characteristics allowed us to identify deficits in EMT and cellular proliferation that may be contributing to the process of abnormal valve remodeling and identify dysregulated signaling pathways that may be investigated as therapeutic targets.

Although other mouse models have been developed that exhibit semilunar valve disease, this is the first to recapitulate semilunar valve stenosis through the incorporation of an orthologous human mutation (reviewed by Gomez Stallons et al., 2016; Wu et al., 2017). Most models of valve stenosis rely upon knocking out gene expression, without considering the biological relevance of such severe genetic manipulations. As most human disease-causing mutations are hypomorphic (not complete loss of function), the incorporation of specific mutations may be an important consideration when exploring the impact of disease-causing mutations. The advent of genome editing has expedited the process of mouse model generation and, as such, should allow for ease in the generation of future models of human disease. This approach permits *in vivo* validation of mutation pathogenicity and supports the identification of disease mechanisms, which can facilitate the identification of future therapeutic targets.

Semilunar valve defects were identified in the developing *Gata4^G295Ski/wt^* mouse model, and include deficits in EMT and cell proliferation, with a subsequent reduction in leaflet volume. Previous work has highlighted an essential role for GATA4 in the process of atrioventricular valve EMT and development, however the role of GATA4 in EMT has not previously been studied in relation to the semilunar valves (Rivera-Felicano et al., 2006). Our findings suggest that EMT defects, due to the *Gata4* p.Gly295Ser mutation, lead to significantly smaller OFT cushions at E13.5, predominantly because of a smaller NCC. This could be the result of a more dramatic effect on the NCC from the EMT deficit, or potential compensation by other cell types in the RCC and LCC. By E15.5, the total volume of the leaflets in *Gata4* mutant mice is no longer statistically significantly different from wild-type controls. The cell density data support that the compensation was at least in part due to presence of increased ECM during valve remodeling. Although leaflet size was compensated for by E15.5, the mutant leaflets were noticeably dysmorphic compared with wild type, which may be because of abnormal ECM. Another possibility is that there are compensating cell types that provide only a partial rescue. Interestingly, our phenotype and proposed disease mechanism are similar to the recently described endothelial deficient *Brg1* mouse model. In the *Brg1* model, EMT is disrupted and it is proposed that this leads to compensation by another cell type, which ultimately results in a stenotic valve (Akerberg et al., 2015). The development of the endocardial cushion into the mature valve leaflet is a complex process involving EMT, CNC invasion and remodeling. Similar to the *Brg1* model, our data suggest that loss of functional GATA4 leads to EMT deficits in the developing OFT and could results in alterations in the contributions of other invading cell types. The significance of the smaller NCC at E13.5 for valve morphology and function in adulthood is unknown. A limitation of our analysis is that we were unable to accurately assess the size of each aortic valve cusp in the adult mouse. Future work involving lineage tracing will help to elucidate the role that EMT disruption in the OFT has on cellular contribution to the semilunar valves.

Molecular profiling of the E15.5 OFTs identified several pathways that were dysregulated, at the RNA level, in the *Gata4^G295Ski/wt^* OFTs. The most significantly dysregulated pathway, identified by KEGG pathway analysis, was the Wnt signaling pathway (Fig. 7). The Wnt signaling pathway has multiple essential roles during cardiac development, including the regulation of EMT and proliferation, as well as CNC migration and later roles in valve maturation and homeostasis (Hurlstone, et al., 2003; Liebner et al., 2004; Alfieri et al., 2010; Bosada et al., 2016; Hulin et al., 2017; Goddard et al., 2017). Our data suggest that the canonical pathway, the Wnt/Ca^2+^ pathway, is dysregulated in the diseased *Gata4^G295Ski/wt^* OFT. In the OFT, canonical Wnt signaling has been shown to play important roles in the in SHF and CNC. Wnt signaling, which signals through β-catenin, is required for EMT in the pOFT (Bosada et al., 2016). Wnt signaling has also been shown to activate TCF-dependent transcription, required for CNCs to undergo G1/S transition and subsequent migration to the OFT (Kirby and Hutson, 2010). We show that β-catenin protein expression is decreased in the *Gata4^G295Ski/wt^* aortic valves compared with wild-type littermate controls. This result is consistent with the recent report demonstrating the importance of canonical Wnt signaling during the later stages of heart valve remodeling driven by hemodynamic forces (Goddard, et al., 2017). It is important to note that the transcriptomic studies were performed at E15.5, which is a post-EMT timepoint. Given the EMT and cell proliferation defects present early in the development of the *Gata4^G295Ski/wt^* aortic valves, the RNA-seq data at E15.5 are most likely identifying compensatory gene expression changes that result from the initial cellular deficits. Accordingly, the dysregulated gene expression that we have identified is most likely secondary, rather than the result of a deficit related to direct transcriptional targets of GATA4. Importantly, this data still provides valuable information and points to potential therapeutic targets that may be harnessed as future therapies to ameliorate the development and progression of valvar stenosis.

The *GATA4* p.Gly296Ser mutation was identified in a large family with inherited atrial septal defect and partially penetrant pulmonic valve stenosis. *GATA4* mutations have been associated with numerous cardiac defects, including atrial septal defect, ventricular septal defect, atrioventricular septal defect, tetralogy of Fallot, persistent truncus arteriosus, patent ductus arteriosus, partial anomalous pulmonary venous return, as well as pulmonic stenosis in the setting of atrial septal defect. Mutations in *GATA4* that are associated with pulmonic stenosis in the setting of an atrial septal defect have been reported in four separate families, three families with p.Gly296Ser mutations and one family with a p.Lys319Glu mutation (Garg et al., 2003; Sarkozy et al., 2005; Xiang et al., 2014). Interestingly, evaluation of the Pediatric Cardiac Genomics Consortium (PCGC) data for 2871 children with CHD only identified a single *de novo GATA4* mutation (Jin et al., 2017). This frame shift mutation was identified in a patient with atrial septal defects, pulmonic valve stenosis and mitral valve disease. Although the *GATA4* p.Gly296Ser mutation results in specific functional deficits based upon our previous studies (Misra et al., 2012), these human genetics findings support that heterozygous loss-of-function of GATA4 contributes to pulmonic valve stenosis. In support of this, examination of a small number of mice heterozygous for the *Gata4*-null allele demonstrated a partially penetrant phenotype of aortic valve stenosis (data not shown). Another interesting aspect from these human genetics reports is the finding of *GATA4* mutations in patients with both pulmonic valve stenosis and an atrial septal defect, supporting the idea that *GATA4* mutations only cause pulmonic valve stenosis in the setting of an atrial septal defect. Perhaps, it is the reduction of EMT in the OFT, along with alterations in intracardiac flow in the developing embryos due to the presence of an atrial septal defect, or deficits in cardiomyocyte proliferation, that together cause this phenotype. Further querying of large databases containing CHD cohorts will be required to determine whether *GATA4* variants are only associated with pulmonic valve stenosis and atrial septal defect phenotypes.

Although our model recapitulates the semilunar valve stenosis phenotype, some questions remain when comparing the mouse phenotype with the human phenotype. One of the key differences is in the affected valves. The aforementioned patients always have an affected pulmonary valve, whereas in the mouse it is usually the aortic valve. Although there is no specific or precise answer as to why this occurs, it may be because of the differences in physiology and anatomy that exist between humans and mice. The blood flow to organs, such as the brain, is different in humans than it is in mice, which could lead to changes in the hemodynamics and pressure that the semilunar valves experience during and after development. In addition, heart rate and cardiac output differ between species. The semilunar valve disease is similar in respect of the cause of the valve stenosis, as it is the result of thickened valve leaflets as opposed to a hypoplastic valve annulus. Although the affected valve in this mutant mouse is predominantly the aortic valve, unlike the affected patients, it is still a valuable model of CHD as it is, to our knowledge, the first model of a human mutation causing congenital valve stenosis in a mouse. In addition, as the semilunar valves are derived from the same populations of cells and undergo very similar molecular signaling patterns, these studies are valuable to the central understanding of the role of GATA4 in semilunar valve development and disease.

In 2017, a genome wide-association study identified an association between a variant in the enhancer of *GATA4*, as well as a near significant coding variant p.S377G, with BAV (Yang et al., 2017). This study subsequently identified a loss of EMT when GATA4 was depleted in induced pluripotent stem cell collagen gel assays. Subsequently, a novel *GATA4* variant that resulted in a stop-gain, p.E147X, was associated with autosomal dominant inheritance of familial BAV (Li et al., 2018). Although congenital valve stenosis and BAV are separate phenotypes, and our model does not have BAV, these findings in addition to our work are indicative of a fundamental role for GATA4 in the developing OFT, highlighting the essential role of GATA4 in EMT.

The *Gata4^G295Ski/wt^* mouse is a novel model for congenital aortic valve stenosis, and is the first valve stenosis model generated by the incorporation of a human mutation. This model allows for the examination of the impact of the hypomorphic *Gata4* p.Gly295Ser allele on the development of the semilunar valves. Through this analysis, we identified a deficit in EMT and subsequent developmental malformations in the developing semilunar valves. GATA4 is a known regulator of EMT and has recently been associated with BAV; these recent findings, combined with our work described here, lend strong support towards a novel and critical role for Gata4 in development and disease of the semilunar valves.

## MATERIALS AND METHODS

### Mouse strains and genotyping

Animal use was approved and monitored on protocol AR09-00040 by the Institutional Animal Care and Use Committee at the Research Institute at Nationwide Children's Hospital. *Gata4^G295Ski/wt^* mice were kept on a C57BL/6J background and genotyped using an allelic discrimination assay (Applied Biosystems), which is able to detect the p.G295S point mutation using fluorescently labeled VIC- and FAM-labeled probes GATA-p.G295SFAM (6FAMTGTAATGCCTGCAGCCMGBNFQ) and GATA-p.G295SVIC (VICAATGCCTGCGGCCTMGBNFQ), and primer sequences FWD 5′ CATCCACTCACCCCATGGA 3′ and REV 5′ CACGCTGTGGCGTCGTAAT 3′, as previously described (Misra et al., 2012).

### Echocardiography

Transthoracic echocardiography was performed with the VisualSonics VEVO 2100 Ultrasound System, as previously described (Misra et al., 2012; Stypmann et al., 2009). The mice were sedated with 3% isoflurane and then titrated to 1-2% isoflurane for maintenance of a heart rate at ∼500 beats per minute. The velocity across the aortic and pulmonic valve were measured using the pulsed-wave Doppler. Analysis was performed in a genotype blind fashion.

### Tissue fixation and histology

For embryonic and adult timepoints, tissues were collected and fixed for at least 24 h in 10% formalin. For calcification studies, tissues were fixed in 4% paraformaldehyde. Tissues were subsequently embedded in paraffin and serial sectioning was performed at a thickness of 6 μm. Staining was performed using Hematoxylin and Eosin (H&E) (Sigma Aldrich), following the manufacturer's protocol. Russell-Movat's Pentachrome staining (American MasterTech, KTRMP) was performed following the manufacturer's protocol. Calcification staining was performed using the Alizarin Red Solution (Millipore, 2003999). All images were visualized using the Zeiss Imager.A2 Microscope and taken on the Zeiss Axiocam MRC r3.1 Camera.

### Cardiac OFT cushion explant assay

Explant assays were performed following a modified protocol by Okagawa et al. (2007). Briefly, pOFTs from E10.5 wild-type and *Gata4^G295Ski/wt^* embryos were excised and placed on hydrated 3D collagen gels (Collagen I, Rat Tail, Corning, 354249). The gels were equilibrated with M199 (Gibco, 11150059) and supplemented with insulin-transferin-selenium (Corning, 25800CR), penicillin/streptomycin (Gibco, 15140122), and fetal bovine serum (Atlanta Biologicals, S11195H). Explants were allowed to attach with the endocardium facing down and were incubated for 48 h at 37°C, followed by imaging and quantification. Quantification was performed in a blinded fashion, and all cells that were emerging from the central mass were counted following a methodology as previously reported (Zhang et al., 2014). Two obvious cellular phenotypes were observed: cells actively undergoing activation and delamination (spindle shaped) and cells that have not undergone transformation (round shaped). The total number of cells was equal to spindle shaped plus round shaped cells.

### Immunostaining

Immunohistochemical detection of GATA4 was performed using anti-GATA4 primary antibody (1:500, Santa Cruz Biotechnology, #SC-1237) followed by biotinylated anti-goat secondary antibody (1:500, Vector Laboratories, BA-9500) and visualized with Vectastain ABC HRP kit (Vector Laboratories, #PK-4000). β-Catenin expression was also examined by immunohistochemistry using anti-β-catenin antibody (1:200, Abcam, #ab16051) and anti-rabbit SignalStain Boost IHC Detection reagent (Cell Signaling Technology, 8114) followed by visualization with Signal Stain DAB Substrate kit (Cell Signaling Technology, 8059). All washing was performed using 1× PBS (for GATA4) or 1× TBS (for β-catenin) containing 0.1% Tween-20. β-Catenin positive staining was quantified using ImageJ. An average of at least two randomly chosen areas in the valve region from each high magnification image were considered for quantification. All images were visualized utilizing the Zeiss Imager.A2 Microscope and taken on the Zeiss Axiocam MRC r3.1 Camera.

### AMIRA 3D reconstruction

AMIRA 3D reconstruction software (version 5.5.0) was used to analyze valve morphology at a 3D level. Briefly, 6 μm serial sections that contained the entire aortic valve were collected. These sections were stained with H&E, histological sections were imaged, aligned with the AMIRA software, and then regions were selected with the software for reconstruction. Volumetric analysis was collected separately for each leaflet using the measure function.

### Cellular apoptosis, proliferation and density

TUNEL assay was performed to detect cellular apoptosis using In Situ Cell Death Detection Kit, Fluorescein (Roche, 11684795910), containing terminal deoxynucleotidyl transferase (TdT) and label solution (fluorescein-dUTP). Briefly, tissue-sections were incubated with TdT enzyme with fluorescein-dUTP to label the nicked DNA of the apoptotic cells following the manufacturer's instructions. For nuclear staining, slides were mounted using Vectashield^®^ Antifade Mounting Medium with DAPI (Vector Laboratories, H-1200). Cellular proliferation was detected using an immunofluorescence technique using rabbit anti-phospho-Histone H3 antibody (1:250, EMD Millipore, 06-570) and donkey anti-rabbit secondary antibody tagged with Alexa Fluor 594 (1:400, Thermo Fisher Scientific, A-21207) following standard protocol and mounted using Vectashield^®^ as previously described (Basu et al., 2017). All images were visualized using an Inverted Epi-Fluorescence Research Microscope (Olympus IX51) equipped with a Cooled CCD Scientific Camera. An average of at least two sections from each embryo were considered for quantification. For the cell density measurements, all of the above-mentioned sections with DAPI staining were used. That number of cells was counted from two randomly chosen areas (298.6 mm^2^ or 40×60 pixels) and averaged for each valve section from three independent *Gata4^G295Ski/wt^* and wild-type littermate embryos.

### RNA-seq

RNA-seq was performed on E15.5 OFTs microdissected in an RNase-free setting and total RNA was collected using the Total RNA Purification kit (Norgen Biotek Corp, 17200). Libraries were generated using the RNA TruSeq Stranded Total RNA LT with Ribo-Zero Gold Set A kit (Illumina, RS-122-2301). Fifty base paired end reads were performed on an Illumina High-Seq 2500. Fastq files were aligned to the mouse genome (mm9) using the STAR aligner version 2.4.0 in two pass mode using default parameters (Dobin et al., 2013). Quality control (QC) was performed on aligned files using RNASeQC and RSeQC to verify alignment quality (De Luca et al., 2012; Wang et al., 2012). Resulting BAM files were processed using cufflinks pipeline version 2.2.0 (cufflinks, cuffdiff, cuffnorm and cuffquant) for gene expression and differential expression (Trapnell et al., 2012). Principal component analysis identified a potential batch effect in sample knock-in 2 (KI2), as it did not group with other knock-in samples, and it was therefore removed from the study (Fig. S7). RNA-seq data has been deposited in GEO under accession number GSE117621.

Expression intensity was log-2-transformed for 457 genes with *P*-values≤0.05 and fold changes greater than 1.25, and a heatmap was generated using Gene Cluster 3.0, with the intensity values adjusted by centering genes. Clustering was performed using centered correlation as a distance measure and average linkage as method. Intensity is on a red (upregulated) to green (downregulated) scale with representative log 2 units. A volcano plot was generated using ggplots2 to represent differential expression between *Gata4^G295Ski/wt^* versus wild-type, and generated as log2fold change versus −log10 *P*-value.

### RNA-seq functional annotation

Functional annotation was performed on 1146 statistically significantly differentially expressed genes (*P*-value≤0.05). DAVID version 6.8 was used to identify enriched GO terms, and the GO-FAT terminology was used to filter out very broad GO terms based on measured specificity (Huang et al., 2009; Walter et al., 2015). The redundancy of identified GO terms was reduced using the GOplot function reduce_overlap, cutoff set to 0.6. Of the resulting terms, the top 100 were plotted using the GOBubble GOplot function, the top 16 most significant terms are labeled 1-16. Of the top 16 GO terms, 10 terms (cardiovascular system development, cell migration, regulation of signaling, biological adhesion, extracellular matrix, embryo development, extracellular exosome, positive regulation of metabolic processes, cell proliferation and cell surface) were highlighted in a circle graph using the GOCircle function. The circle plot highlights the upregulated (red dots) and downregulated (blue dots) genes, the *P*-value (height of center squares), and *z*-score (color of center squares). The *z*-score is a measurement used to predict whether a term is upregulated or downregulated and is calculated by counting the number of upregulated statistically significant genes in each term, subtracting the number of downregulated genes, and dividing by the square root of the number of genes in each term. A chord plot was generated using the GOChord function, and highlights the association of 45 differentially expressed genes and their association to the following significant GO terms: cardiovascular system development, cell migration, extracellular matrix, biological adhesion and extracellular exosome. In addition, the chord plot highlights the log fold change (logFC) associated with each gene on a scale of red (upregulated) to blue (downregulated).

KEGG Pathway analysis was also performed using DAVID version 6.8, and the top six statistically significant KEGG pathways are represented as a circle plot using the GOCircle function, as previously described (Kanehisa and Goto, 2000 ;Walter et al., 2015). A chord plot was generated using the GOChord function, as previously described, and highlights the association of 40 significantly differentially expressed genes and their association with the following significant GO terms: Wnt signaling, axon guidance, focal adhesion, ECM-receptor interaction and PI3K-AKT signaling pathway. Wnt signaling, the most significantly enriched KEGG pathway, is shown with differentially expressed genes highlighted with a red star (downregulated) or yellow star (upregulated).

### RNA purification and RT-qPCR

RNA was extracted from microdissected E15.5 OFTs in an RNase-free setting. Briefly, OFT tissues were harvested in TRIzol, and homogenized using TissueLyser II (Qiagen), followed by chloroform extraction and purification using the Total RNA Purification kit (Norgen Biotek Corp, 17200) following manufacturer's instruction. RNA was quantified spectrophotometrically (Thermo Fisher Scientific, NanoDrop 2000), and 500 ng of total RNA was used for reverse transcription using the SuperScript VILO cDNA Synthesis Kit (Thermo Fisher Scientific, 11754-050). SYBR Green based RT-qPCR was performed using Applied Biosystems 7500 Fast machine. The candidate genes and oligonucleotide sequences are listed in Table S2. Mean relative gene expression was calculated after normalizing C_t_ values to endogenous *Gapdh* control, using the ^ΔΔ^Ct method. Three independent experiments were performed using three sets of *Gata4^G295Ski/wt^* and wild-type littermate controls, and each set consists of a pool of three E15.5 embryos.

### Statistics

Statistical analysis was performed using Graphpad Prism version 7 using Student's *t*-test, for which a *P*-value≤0.05 was considered significant. An *n* of at least 3 was used for all experiments, the *n* is noted either within the figure legend or in the text for each experiment. Data are mean±s.d.

## Supplementary Material

Supplementary information
