# Supplementary material for: Developmental origins for semilunar valve stenosis identified in mice harboring congenital heart disease-associated GATA4 mutation
Source: Dis Model Mech. 2019 Jun 24;12(6):dmm036764. doi: 10.1242/dmm.036764 (PMC6602309; doi:10.1242/dmm.036764)
Supplement: Supplementary information [file dmm-12-036764-s1.pdf]

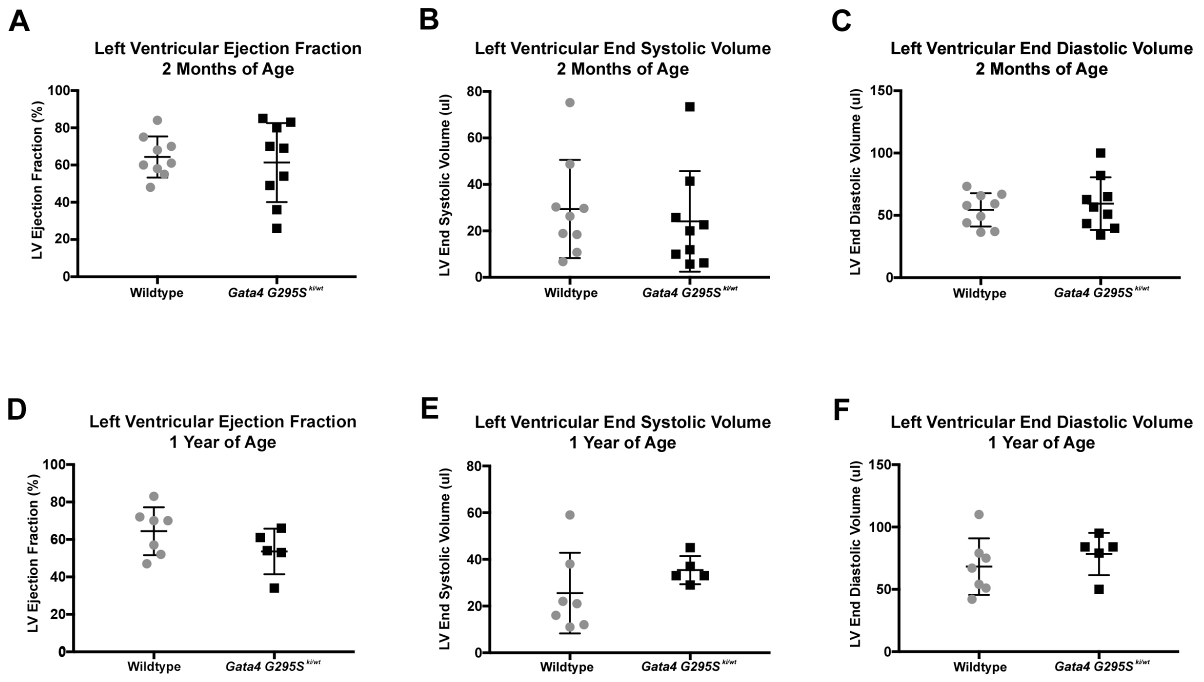

Figure S1. ***Echocardiographic analysis of left ventricular function at one year and two months of age.*** Left ventricular function of *Gata4 G295S*$^{ki/wt}$ and wildtype littermate controls at 2 months **(A-C)** and one year **(D-F)** of age was not found to be significantly different. **A)** and **D)** represent left ventricular ejection fraction, **B)** and **E)** represent left ventricular end systolic volume, and **C)** and **F)** represent left ventricular end diastolic volume.

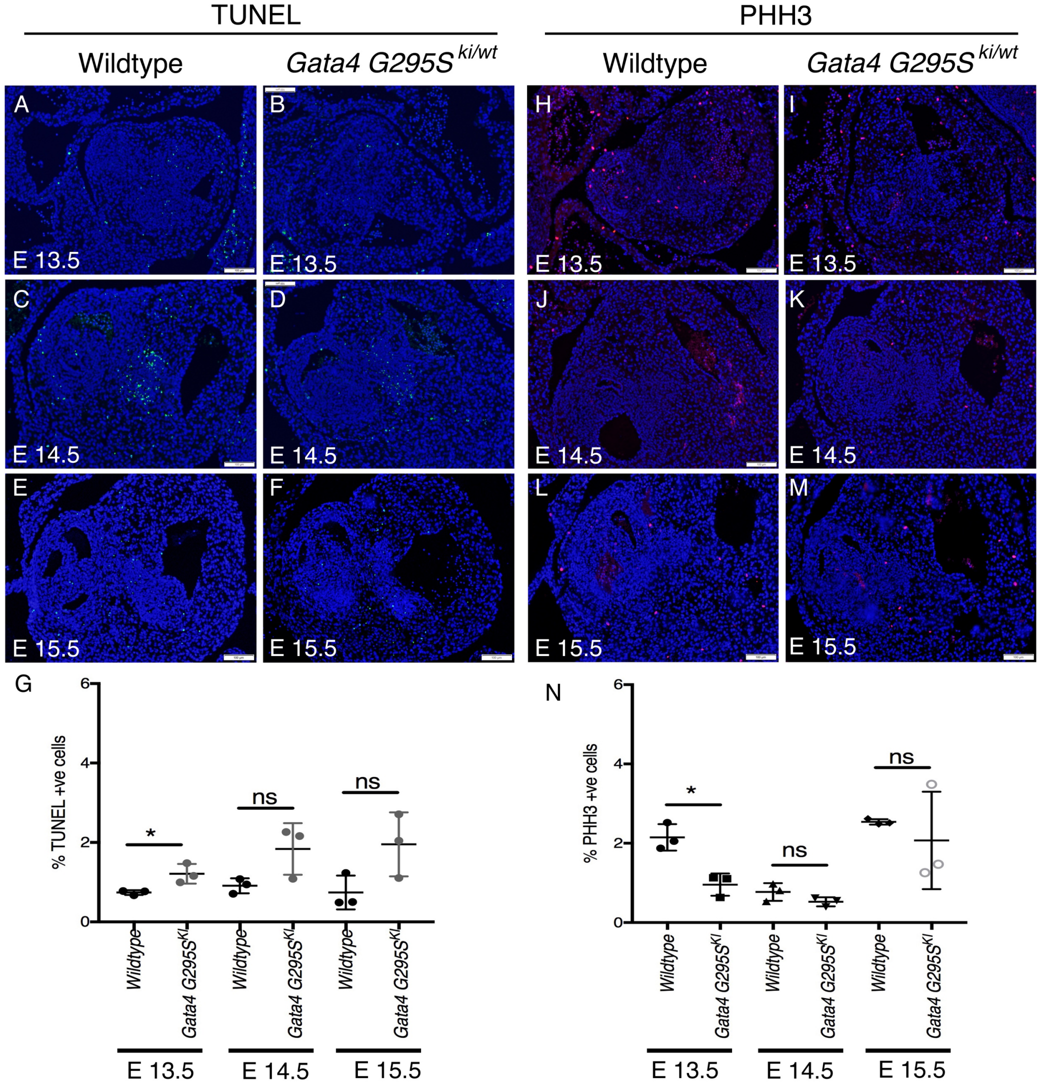

Figure S2. Quantification of cardiac apoptosis and proliferation in cardiac outflow tract cushions in *Gata4*$^{G295Ski/wt}$ and wildtype littermates. Representative images of TUNEL (A-F) and phosphohistone H3 (PHH3) staining is shown from E13.5, E14.5, and E15.5. Quantification of TUNEL and PHH3 staining is shown as a percentage of positive stained cells in valve area in G and H, respectively. Wildtype n=3, *Gata4*$^{G295Ski/wt}$ n=3 for each timepoint; *, p value < 0.05; ns, p value > 0.05.

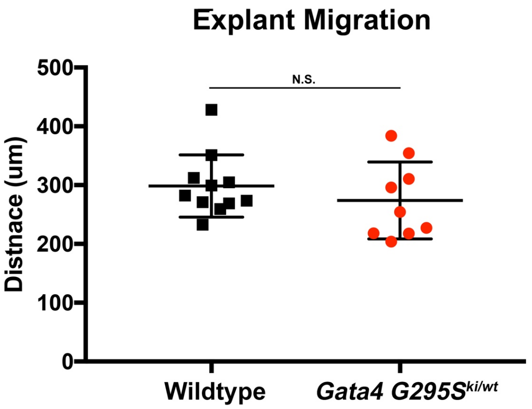

Figure S3. Quantification of migration in OFT explant assay shows no significant differences between *Gata4 G295S$^{ki/wt}$* and wildtype OFTs*.* Averages of 3 measurements of distance per explant were assessed. Black boxes represent wildtype measurements and red circles are *Gata4$^{G295Ski/wt}$* measurements. N.S. = p value > 0.05.

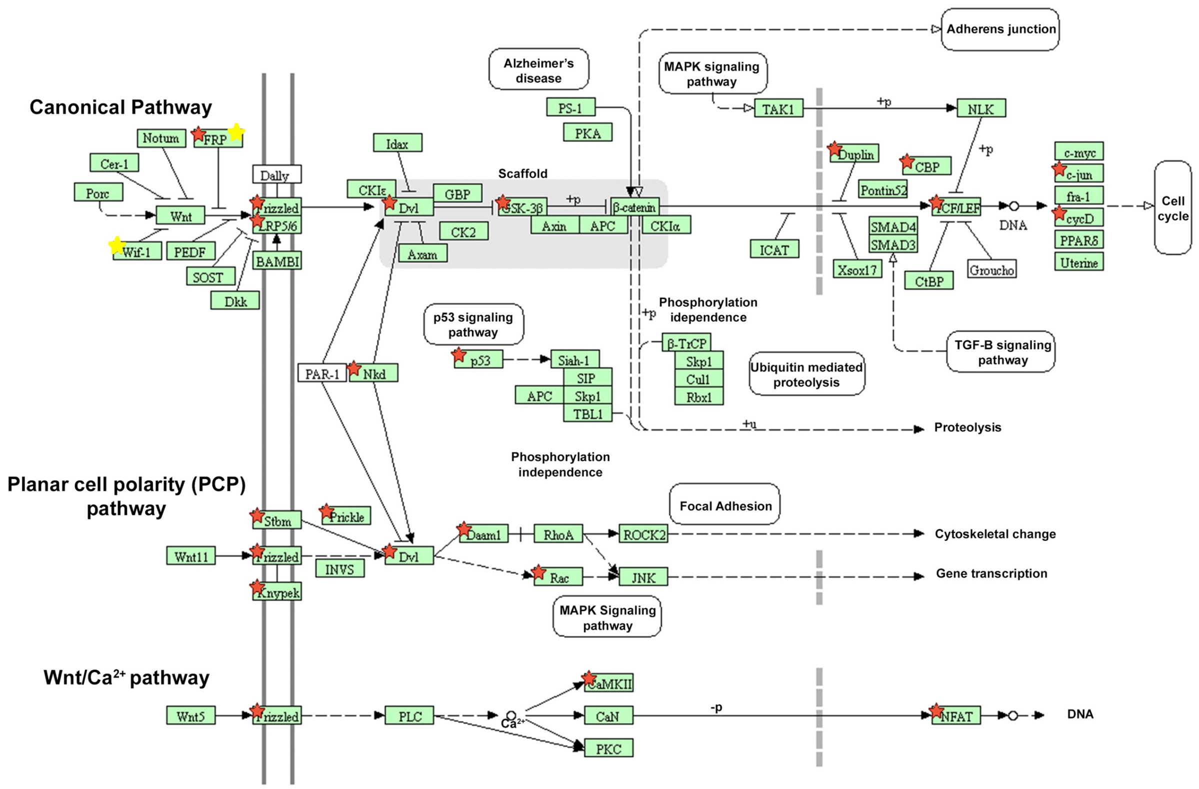

Figure S4. Dysregulation of Wnt signaling in *Gata4*$^{G295Ski/wt}$ outflow tracts by RNA-seq. The Wnt signaling KEGG pathway exhibits multiple components differentially expressed in the *Gata4*$^{G295Ski/wt}$ OFTs compared to wildtype littermate control. Red (downregulated) and yellow (upregulated) asterisks indicate differentially expressed gene. KEGG Copyright permission obtained from Kanehisa and Goto, 2000.

Disease Models & Mechanisms • Supplementary information

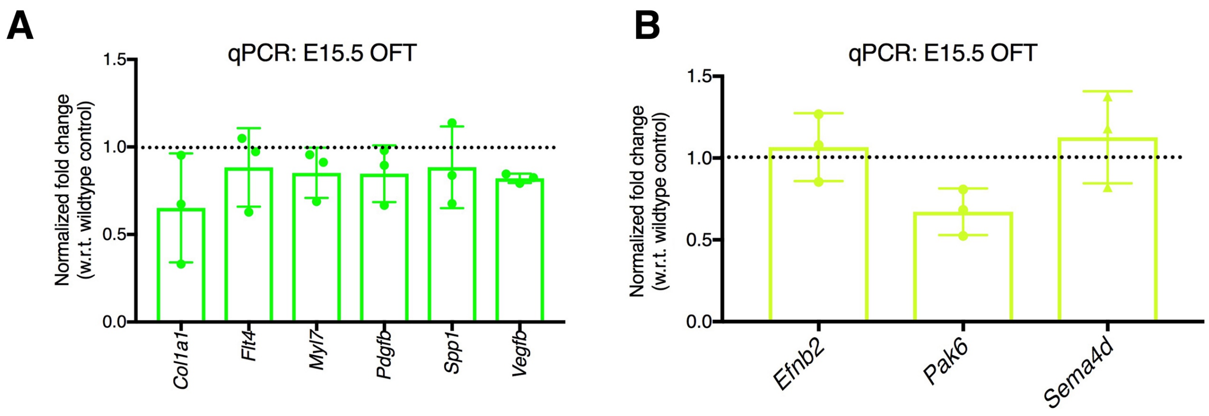

Figure S5. Validation of gene expression changes in axonal guidance and focal adhesion pathways by RT-qPCR. (A) Transcript levels of representative dysregulated genes in the focal adhesion pathway by RT-qPCR measured in E15.5 *Gata4*$^{G295Ski/wt}$ OFTs by RT-qPCR when compared to wildtype littermate OFTs (n=3). (B) Transcript levels of dysregulated genes in axon guidance pathway by RT-qPCR measured in E15.5 *Gata4*$^{G295Ski/wt}$ OFTs by RT-qPCR when compared to wildtype littermate OFTs (n=3). All genes were normalized with respect to (w.r.t) internal *gapdh* control and dotted line represent wild type expression.

Disease Models & Mechanisms • Supplementary information

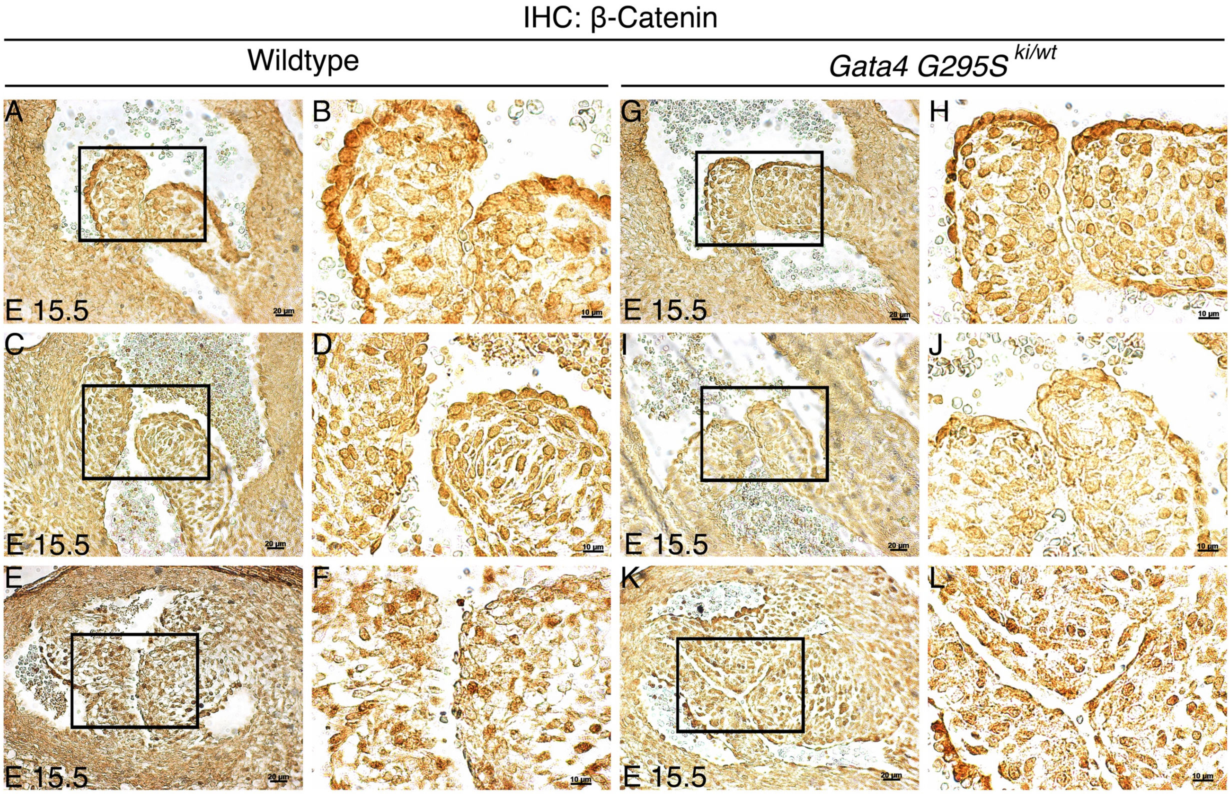

Figure S6. Decreased expression of beta-catenin in E15.5 ***Gata4$^{G295Ski/wt}$*** **aortic valve leaflets.** Immunohistochemistry for β-catenin protein in E15.5 aortic valve leaflets from *Gata4$^{G295Ski/wt}$* (G-L) and wildtype (A-F) littermates shows an overall decrease in β-catenin expression in mutants, which is more predominant in the interstitial cells but is also seen in the endothelial lining of the developing leaflets. Representative images from three mutant and three wildtype littermate embryos are shown. Blackboxes indicate the high magnification area presented right to each image.

Scale bar represents 20 μm in A, C, E, G, I, K and 10 μm in B, D, F, H, J, L.

Disease Models & Mechanisms • Supplementary information

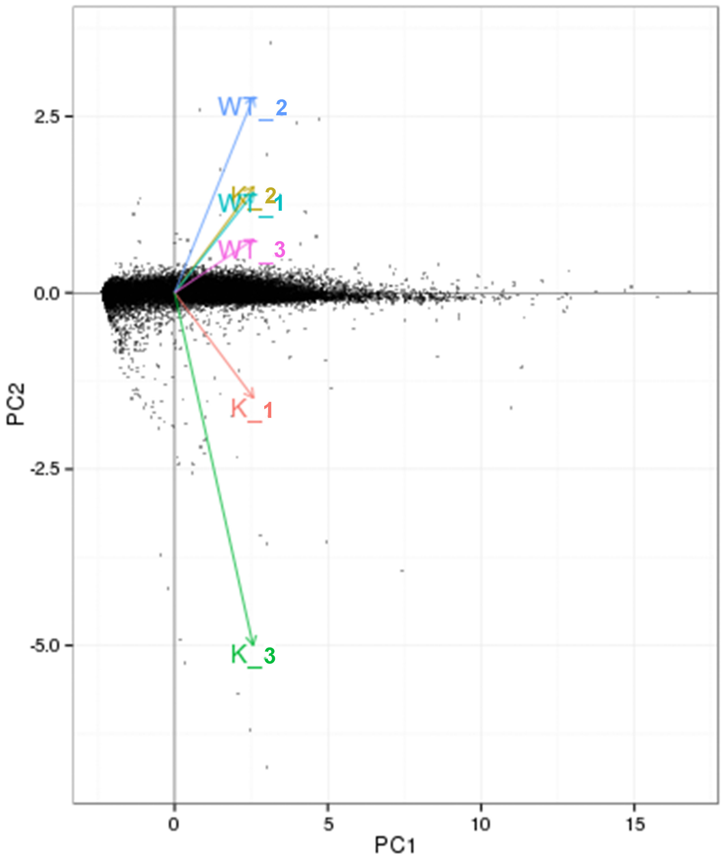

Figure S7. ***Principal Component Analysis of RNA-seq samples.*** Utilization of 2 principle components (PCs) to assess overall differences between RNA-seq samples. Knock-in 2 (yellow) grouped with the wildtype samples (teal, blue, pink), not with the other knock-in samples (red, green), and was removed from the subsequent analysis.

Reference

Kanehisa, M. and Goto, S. (2000). KEGG: kyoto encyclopedia of genes and genomes. *Nucleic Acids Res* **28**, 27-30.

Disease Models & Mechanisms • Supplementary information

Sample	KI Read Depth	WT Read Depth	Variant Frequency
Gata4 G295S$^{ki/wt}$ 1	14	25	36.00%
Gata4 G295S$^{ki/w}$ 3	9	12	43.00%
Wildtype 1	0	44	0.00%
Wildtype 2	0	44	0.00%
Wildtype 3	0	28	0.00%

Table S1. *Gata4* G295S allele frequency in RNA-seq datasets. KI: number of sequencing reads that covered the G295S mutation, WT: number of sequencing reads that read the G295 location as wildtype.

Disease Models & Mechanisms • Supplementary information

Table S2

Gene Symbol	Primer Name	Primer sequence
Ccnd2	CCND2_L	GCCAAGATCACCCACACTGA
	CCND2_R	GCGTTATGCTGCTCTTGACG
Crebbp	CREBBP_L	GCAGGAGGCATGACCAAGAT
	CREBBP_R	ATCTGCTGCCCTCCAGTTTG
Daam2	DAAM_L	TGGCCCCACTGGAAAACTTT
	DAAM_R	CCGAAACTTCTCTGCCTGGT
Dvl3	DVL_L	CGGCCATCGTAAAAGCCATG
	DVL_R	CCGAGCCGATGAAAGCATTG
Fzd2	FZD2_L	TCCTCACATGGTCGGTGTTG
	FZD2_R	TGTAGCAGCCGGACAGAAAG
Fzd10	FZD10_L	TGGGCAGCATGGATGTCAAT
	FZD10_R	AAAGCCGGACAGGATGAAGG
Gsk3b	GSK3B_L	ACCGAGAACCACCTCCTTTG
	GSK3B_R	TGTGGTTACCTTGCTGCCAT
Lrp5	LRP5_L	AAGACAGGGGCTGAGGAAGT
	LRP5_R	TGAAGTCAGGGGTGTCCAGA
Nfatc1	NFATC1_L	GGTGCTGTCTGGCCATAACT
	NFATC1_R	TCCCGGTCAGTCTTTGCTTC
Nfatc2	NFATC2_L	TCGTAGGCAACACCAAGGTC
	NFATC2_R	TGTTCTTCCTGCCGATGTCC
Rac3	RAC3_L	GAAGCTGGCACCCATAACCT
	RAC3_R	AGAGCTGAGCACTCCAGGTA
Sfrp2	SFRP2_L	CAGGACAACGACCTCTGCAT
	SFRP2_R	TCACACACCTTGGGAGCTTC
Tcf7l2	TCF7L2_L	TCGTCACACCGACAGTCAAG
	TCF7L2_R	GGGCTTCTTTATGTGGGGCT
Wif1	WIF1_L	AGAAAGCCCTGTGCATACCC
	WIF1_R	CAGGTGGTTGAGCAGTTTGC
Col1a1	COL1A1_L	CAGGCTGGTGTGATGGGATT
	COL1A1_R	CTCCATCTTTGCCAGCAGGA
Flt4	FLT4_L	AGAATGACCTGGGCCCCTAT
	FLT4_R	TCCTTTGAGCCACTCGACAC
Myl7	MYL7_L	AGCTCGGGAGGGTAAGTGTT
	MYL7_R	GTCCGTCCCATTGAGCTTCT
Pdgfb	PDGFB_L	AGCCCATCTTCAAGAAGGCC
	PDGFB_R	AATGGTCACCCGAGCTTGAG
Spp1	SPP1_L	AATCTCCTTGCGCCACAGAA
	SPP1_R	CATCGTCGTCCATGTGGTCA
Vegfb	VEGFB_L2	GCCAATGTGAATGCAGACCA
	VEGFB_R2	TGATGTCAGCTGGGGAGGAT
Efnb2	EFNB2_L	TCCAACAAGACGTCCAGAGC
	EFNB2_R	CTGTTGCCATCGGTGCTAGA
Pak6	PAK6_L	ACTACCAGCACCTCAACGTG
	PAK6_R	ACAGTGGCAATCTGCTCCTC
Sema4d	SEMA4D_L	TAGTGTTGAGAACCGCGGTG
	SEMA4D_R	GGCTTGTGAAACTGCACCAG

Disease Models & Mechanisms • Supplementary information
